# Coexistence of Cold Agglutinin and Cryoglobulin in a Patient With Severe Anemia Following COVID-19 Infection

**DOI:** 10.7759/cureus.75257

**Published:** 2024-12-07

**Authors:** Masahiko Kaneko, Yuya Masuda, Kenichi Ishikawa, Hisaharu Shikata

**Affiliations:** 1 Department of Internal Medicine, Uwajima City Hospital, Uwajima, JPN; 2 Department of Hematology, Uwajima City Hospital, Uwajima, JPN; 3 Department of Neurology, Uwajima City Hospital, Uwajima, JPN

**Keywords:** cold agglutinin, cold-related hematologic disorders, covid-19, cryoglobulin, direct antiglobulin test, folate deficiency

## Abstract

We report a case of coexisting cold agglutinin and cryoglobulin in a patient with severe anemia following COVID-19 infection, in whom direct antiglobulin testing revealed C3d positivity and immunoglobulin G negativity. There was no evident hemolytic anemia, thrombosis, or clinically significant IgM monoclonal gammopathy. The anemia improved with folic acid supplementation alone accompanied by a decrease of the cold agglutination titer, and the direct antiglobulin test became negative. Despite continuous positivity for cryoglobulinemia, the patient exhibited no clinical symptoms such as thrombosis. Immunosuppressive therapy for cold-related hematologic disorders after COVID-19 infection should be considered carefully.

## Introduction

Previous case reports have described patients with COVID-19-associated autoimmune hemolytic anemia (AIHA), which is recognized as a rare complication [1,2]. Cold agglutinin syndrome (CAS) secondary to COVID-19 infection, characterized by C3d positivity and immunoglobulin G (IgG) negativity by the direct antiglobulin test (DAT), is even rarer [2,3]. Most reported cases have been treated promptly with immunosuppressive agents, including corticosteroids, making it uncertain whether this condition is a transient response or a persistent event requiring treatment. On the other hand, positive DAT results, with or without clinically evident anemia, have been reported in a subset of patients with various viral infections [4]. A higher rate of DAT positivity among COVID-19 transfusion candidates has been reported, whereas hemolysis is very rare [5]. These data indicate that DAT positivity is related to SARS-CoV-2 infection, which is associated with an increased frequency of anemia and a greater requirement for transfusion. However, the mechanism responsible for this association is not well understood.

Cryoglobulins are immunoglobulins that precipitate at low temperatures; their presence in the blood is called cryoglobulinemia, and when they produce symptoms, the term cryoglobulinemia syndrome is used [6]. One of the causes of cryoglobulinemia is viral infection, but only one case of cryoglobulinemic purpura associated with COVID-19 infection has been reported [7], and the relationship between the two remains unclear.

We herein report a case of severe anemia with DAT positivity for C3d but negativity for IgG, along with positivity for cold agglutinin and cryoglobulinemia secondary to COVID-19 infection, which was notably improved by folate supplementation.

## Case presentation

**Figure 1:**
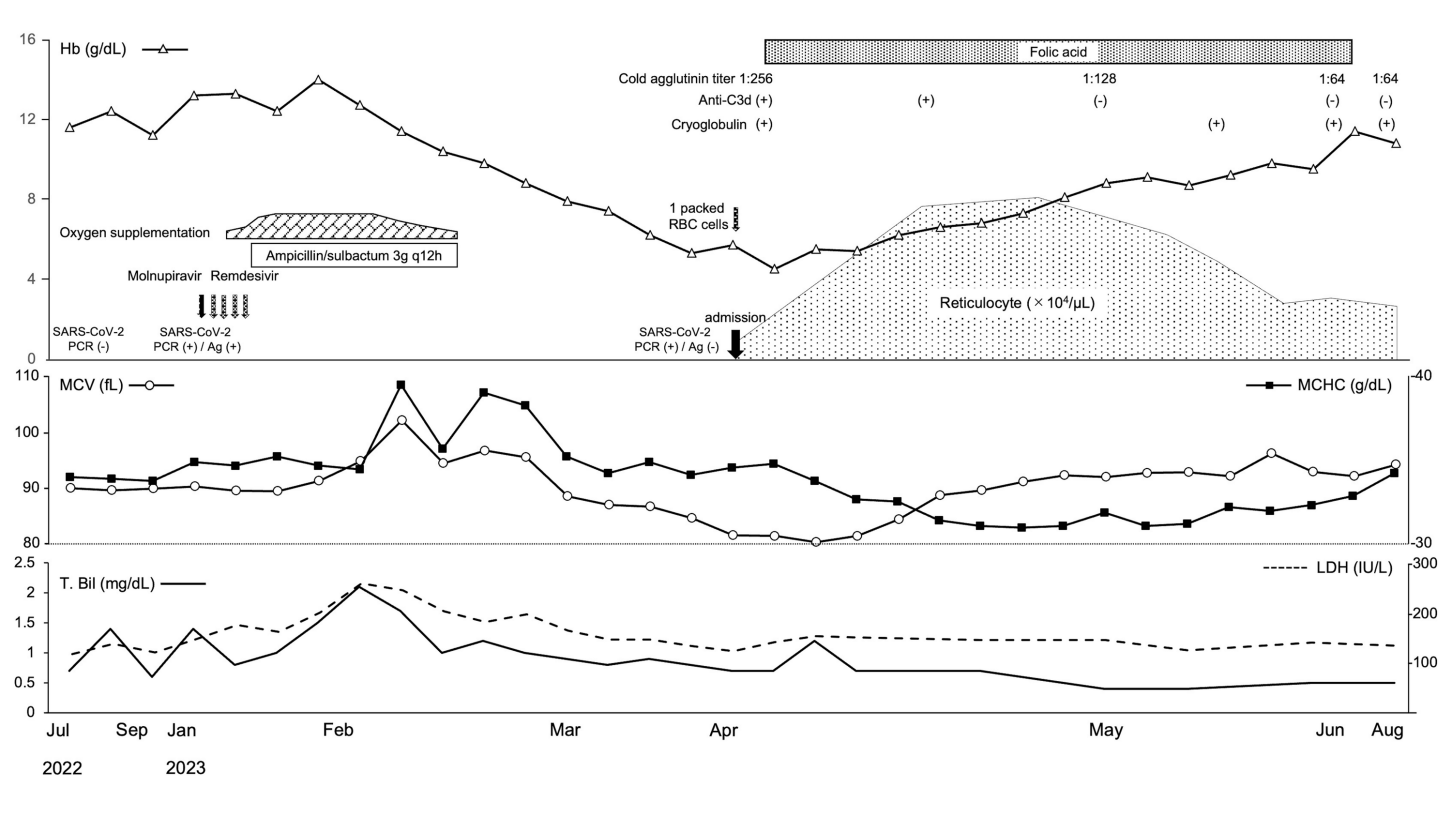
Clinical course. Hb: hemoglobin, DAT: direct antiglobulin test, RBC: red blood cell, SARS-CoV-2: severe acute respiratory syndrome coronavirus 2, PCR: polymerase chain reaction, MCV: mean corpuscular volume, MCHC: mean corpuscular hemoglobin concentration, T-Bil: total bilirubin, LDH: lactate dehydrogenase

In April 2023, a 73-year-old Japanese male patient who had been treated at a local hospital for 16 months was referred to our hospital because of progressive anemia. His medical history included cerebral infarction and vascular cognitive impairment two years earlier, but no known risk factors for cold agglutinin. Two months before referral, he had been diagnosed as having COVID-19 by a nucleic acid amplification test (transcription reverse-transcription concerted reaction method). Nineteen days after the diagnosis of COVID-19, the patient developed an unnaturally elevated serum total bilirubin (T. Bil) level (2.1 mg/dL; normal range: 0.2-1.2 mg/dL) with a direct bilirubin level of 0.5 mg/dL (normal range: 0.0-0.4 mg/dL) and lactate dehydrogenase (LDH) level of 295 U/L (normal range: 122-228 U/L). Additionally, increases in both the mean corpuscular volume (MCV) (102.1 fL; normal range: 80.0-100.0 fL) and mean corpuscular hemoglobin concentration (MCHC) (39.6 g/dL; normal range: 31.0-36.0 g/dL), along with a reduced red blood cell (RBC) count, were evident, followed by progressive anemia (Figure 1).

Due to the lack of facilities at the referring hospital, it was not possible to prepare a blood smear specimen to confirm the presence of polychromasia or spherocytes. In addition, testing of reticulocyte and serum haptoglobin levels and the Coombs test were not performed. The patient’s hemoglobin level decreased progressively, and he was referred to our hospital 40 days after COVID-19 infection. 

On admission, a complete blood count revealed microcytic normochromic anemia with an Hb level of 5.7 g/dL, MCV of 86.3 fL, MCHC of 35.2 g/dL, and a reticulocyte count of 1.64 × 10^4^/µL (normal range: 4.00-8.00 × 10^4^/µL). White blood cell count fractions and platelet counts were normal. Serum indirect Bil, LDH, haptoglobin, iron, ferritin, total iron binding capacity, vitamin B12, zinc, and copper levels were also all normal. However, a low level of folate (0.6 ng/mL; normal range 3.6-12.9 ng/mL) was confirmed. The immunoglobulin level and complement components were within the normal ranges (Table 1).

**Table 1:** Laboratory findings on admission. WBC: white blood cells, RBC: red blood cells, MCV: mean corpuscular volume, MCH: mean corpuscular hemoglobin, MCHC: mean corpuscular hemoglobin concentration, T-Bil: total bilirubin, Ind-Bil: indirect bilirubin, AST: aspartate aminotransferase, ALT: alanine aminotransferase, LDH: lactate dehydrogenase, ALP: alkaline phosphatase, GGT: γ-glutamyltransferase, TP: total protein, ALB: albumin, BUN: blood urea nitrogen, Cre: creatinine, CRP: C-reactive protein, sIL-2R: soluble interleukin 2 receptor alpha, Fe: iron, TIBC: total iron binding capacity, ANA: antinuclear antibody, Ab: antibody, LA-DRVVT: lupus anticoagulant dilute Russell viper venom time, CL-β2GP1: cardiolipin beta2 glycoprotein I, FLC: free light chain, IE: immunofixation electrophoresis, Ag: antigen, HB: hepatitis B, HCV: hepatitis C virus, HIV: human immunodeficiency virus, EBV: Epstein-Barr virus, EBNA: Epstein-Barr virus nuclear antigen

Parameters	Normal range	Patient results	Parameters	Normal range	Patient results
WBC	4.0–9.0 ×10^3^/μL	5.64	Fe	64–187 μg/dL	146
RBC	450–510 ×10^4^/μL	200	Ferritin	30.0–400.0 ng/mL	480.0
Hematocrit	39.0–52.0 %	16.3	TIBC	250–410 μg/dL	165
Hemoglobin	12.0–16.0 g/dL	5.7	Haptoglobin	19–170 mg/dL	178
MCV	90.0–105.0 fL	81.5	Vitamin B12	233–914 pg/mL	797
MCH	27.0–32.0pg	28.7	Folic acid	3.6–12.9 ng/mL	0.6
MCHC	28.0–36.0 g/dL	35.2	Zinc	80–130 μg/dL	73
Reticulocyte	0.5–2.0 %	0.82	Copper	68–128 μg/dL	83
Platelet	15.0–45.0×10^4^/μL	41	Cold agglutinin	1:64	1:256
T-Bil	0.2–1.2 mg/dL	0.7	Cryoglobulin	(-)	(+)
Ind-Bil	0.2–0.9 mg/dL	0.4	ANA	1:40	1:40
AST	13–33 IU/L	12	anti-double stranded DNA Ab	0.0–9.9 IU/mL	2.1
ALT	8–42 IU/L	13	anti-Ro/ SSA Ab	0.0–6.9 U/mL	1.2
LDH	124–222 IU/L	118	anti-La/SS-B Ab	0.0–6.9 U/mL	< 0.4
ALP	38–113 IU/L	70	anti-U1RNP Ab	0.0–4.9 U/mL	0.8
GGT	11–58 U/L	15	LA-DRVVT	0–1.3 ratio	0.9
TP	6.6–8.7 g/dL	6.4	CL-β2GP1	0–3.5 U/mL	< 1.3
Alb	3.4–4.8g/dL	2.9	FLC κ chain	3.3–19.4 mg/L	102
BUN	5–23 mg/dL	9	FLC λ chain	5.7–26.3 mg/L	48.3
Cre	0.36–1.06 mg/dL	0.44	FLC κ/λ ratio	0.26–1.65	2.11
CRP	0.0–0.3 mg/dL	1.41	IE M-protein	(-)	(-)
IgG	680–1620 mg/dL	1563	HBs Ag	(-)	(-)
IgA	84–438 mg/dL	612	HCV Ab	(-)	(-)
IgM	57–288 mg/dL	56	HIV Ab	(-)	(-)
C3	65–135 mg/dL	79	Mycoplasma pneumoniae Ab	1:40	1:40
C4	13–35 mg/dL	15	EBV anti-VCA IgG	1:10	1:320
CH50	30–45 U/mL	28	EBV anti-VCA IgM	1:10	1:10
sIL-2R	122–496 U/mL	845	EBNA	1:10	1:80

The DAT with monospecific antisera showed positivity for C3b/C3d and negativity for IgG. This patient’s sera agglutinated all of a commercial panel of reagent RBCs in saline agglutination tests at 4°C, but not in the antiglobulin phase. No antibody specificity could be identified (Table 2).

**Table 2:** Results of RBC agglutination assays. DAT: direct antiglobulin test

Parameters	Patient results
Irregular antibodies	
25 ℃	Weekly positive
anti-M	Positive
4 ℃	Strong positive
37 ℃	Negative
DAT with broad spectrum antisera	Positive
DAT with monospesific antisera	
IgG	Negative
C3bC3d	Positive
C3d	Positive

The cold agglutinin titer was 1:256 and the cryoglobulin test gave a positive result. Serum protein electrophoresis, immunofixation electrophoresis, and serum-free light chain were unremarkable. Bone marrow aspiration and biopsy, flow cytometry, and testing for immunoglobulin heavy chain rearrangement showed no evidence consistent with clonal B-cell expansion. Serological tests for Epstein-Barr virus, cytomegalovirus, human immunodeficiency virus, mycoplasma, hepatitis B and C, and autoantibodies associated with connective tissue diseases were all negative (Table 1).

A computed tomography scan of the neck, chest, and abdomen demonstrated no abnormality. 

After admission, one pack of red blood cells was transfused, followed by oral folate supplementation alone, and the anemia improved. Two months later, the cold agglutinin titer decreased to 1:64 and the result of the direct antiglobulin test changed to negative (Figure 1). However, despite continuously testing positive for cryoglobulinemia, there are no clinical symptoms such as thrombosis, and the patient remains asymptomatic.

## Discussion

Accumulated case reports and small case series have suggested that AIHA may be associated with COVID-19. Among them, several studies of secondary CAS associated with COVID-19 infection, characterized by a positive DAT result for C3d, have demonstrated hemolytic anemia and, albeit rarely, clotting [8,9]. On the other hand, there has been one case of histologically proven cryoglobulin syndrome after COVID-19 infection, showing membranous proliferative glomerulonephritis, leukocytoclastic vasculitis, and sensorimotor axonal polyneuropathy [7]. 

In the present case, transient elevations in MCV and MCHC, along with a markedly reduced RBC count, as well as increased levels of serum T. Bil and LDH, were observed following COVID-19 infection, which subsequently led to progressive anemia. The presence of cold agglutinin antibodies reacting at 4°C and IgG-negative C3b/C3d-opsonized red blood cells was evident after transfer to our hospital. These findings suggest that erythrocyte aggregation is caused by cold agglutinin, which is active at room temperature, and the subsequent hemolytic reaction may have been present immediately after COVID-19 infection. However, the cause of subsequent anemia progression is difficult to explain by hemolysis. 

Not all patients with virus infections who develop secondary CAS will have clinically significant hemolysis [10]. Berzuini et al. reported that the prevalence of direct Coombs test positivity among transfusion candidates with COVID-19 was about one-half, whereas hemolysis was very rare [5]. They also noted that the serologic features of DAT reactivity in COVID-19 patients differed from those typically observed in AIHA. In their report, no patient serum or eluates (i.e., solutions of antibodies recovered from the RBC surface of DAT-positive COVID-19 patients) were reactive in the indirect antiglobulin test against a commercial panel of reagent RBCs, or with RBCs from healthy donors. However, all eluates containing IgG tested positive with a panel of RBCs prepared from DAT-negative COVID-19 patients. Based on these data, they hypothesized that one possible interpretation of the mechanism underlying DAT reactivity is that hyperinflammation in COVID-19 enhances the deposition of complement C3 and the binding of IgG autoantibodies to RBC membranes, thus promoting the clearance of damaged RBCs by macrophages. However, their report included two patients (4%) who were DAT-positive for C3d only and negative for IgG, but there was no description of cold-mediated IgM autoantibody involvement. Interestingly, Imoto et al. reported two cases of high cold agglutinin titer in patients with COVID‐19 infection without hemolytic anemia and clotting [11]. One of the two patients had macrocytic anemia with RBC agglutination and a low level of zinc, but no evidence of hemolysis. This condition improved with zinc supplementation. Serum from two patients agglutinated adult RBCs at 4°C, similar to our observations in the present case, indicating that the IgM autoantibodies in the patient’s serum could agglutinate all of a commercial panel of reagent RBCs. This indicates that COVID-19 patients with anemia who have cold agglutinins and are positive for C3d on DAT do not necessarily have associated extravascular hemolysis. However, we consider that the severe reduction in the red blood cell count in this case is likely multifactorial, with folate deficiency playing a significant role. Folate deficiency can impair DNA synthesis, disrupt the maturation and division of red blood cells, and ultimately contribute to a reduced red blood cell count in the peripheral blood.

In the present case, cryoglobulin testing was positive but there were no symptoms suggestive of cryoglobulin vasculitis. In addition, it was considered type III cryoglobulinemia, involving polyclonal immunoglobulins, was present because there was no evidence of monoclonal immunoglobulins. Moyano et al. reported a case of mixed cryoglobulinemia following COVID-19 infection [7]. However, their report did not include any description of monoclonal immunoglobulin, leaving the type of cryoglobulinemia unclear. The symptoms of mixed cryoglobulinemia, including type III cryoglobulinemia, are usually referred to as the “Meltzer triad”, consisting of purpura, arthralgia, and weakness [12]. Because these symptoms are nonspecific, they may have been overlooked in our patient. 

## Conclusions

It is unclear whether COVID-19 infection triggers an immune response in the host organism or whether the imbalance of immune tolerance is related to the formation of cryoproteins such as CG and CA. However, such cryoproteins do not necessarily cause clinically significant hemolysis or thrombosis. Therefore, any indication for immunosuppressive therapy should be considered carefully. The present case highlights the importance of understanding the underlying causes and consequences of cold-related hematologic disorders following COVID-19 infection, as well as choosing optimal medical interventions.
